# The association between albumin levels and survival in patients treated with immune checkpoint inhibitors: A systematic review and meta-analysis

**DOI:** 10.3389/fmolb.2022.1039121

**Published:** 2022-12-02

**Authors:** Deniz Can Guven, Taha Koray Sahin, Enes Erul, Alessandro Rizzo, Angela Dalia Ricci, Sercan Aksoy, Suayib Yalcin

**Affiliations:** ^1^ Department of Medical Oncology, Hacettepe University Cancer Institute, Ankara, Turkey; ^2^ Department of Internal Medicine, Hacettepe University Faculty of Medicine, Ankara, Turkey; ^3^ Struttura Semplice Dipartimentale di Oncologia Medica per La Presa in Carico Globale Del Paziente Oncologico “Don Tonino Bello”, Bari, Italy; ^4^ Medical Oncology Unit, National Institute of Gastroenterology, “Saverio de Bellis” Research Hospital, Castellana Grotte, Italy

**Keywords:** biomarker, cancer, immunotherapy, prognosis, albumin

## Abstract

**Background:** The albumin levels may potentially be used as a prognostic biomarker in patients with cancertreated with immune checkpoint inhibitors (ICIs) due to its close relationship with nutritional and inflammatory status. However, the available data is limited with heterogeneous patient cohorts, sample sizes and variable cut-offs. Therefore, we conducted a systematic review and meta-analysis to evaluate the association between survival outcomes and albumin levels in patients treated with ICIs.

**Methods:** We conducted a systematic review using the PubMed, Web of Science, and Embase databases to filter the published studies up to 1 June 2022. The meta-analyses were performed with the generic inverse-variance method with a random-effects model due to the high degree of heterogeneity. The primary outcome measure was hazard ratio (HR) with 95% confidence intervals (CI). The study protocol was registered with the PROSPERO registry (Registration Number: CRD42022337746).

**Results:** Thirty-six studies encompassing 8406 cancer patients with advanced disease were included in the meta-analyses. Almost half of the studies were conducted in NSCLC cohorts (n = 15), and 3.5 gr/dL was the most frequently used albumin cut-off in the included studies (n = 20). Patients with lower albumin levels had a significantly increased risk of death (HR: 1.65, 95% CI: 1.52–1.80, *p* < 0.0001) than patients with higher albumin levels. Subgroup analyses for study location, sample size, tumor type and albumin cut-off were demonstrated consistent results. Furthermore, in the subgroup analysis of eight studies using albumin levels as a continuous prognostic factor, every 1 gr/dL decrease in albumin levels was associated with significantly increased risk of death by a factor of 10% (HR: 1.10, 95% CI: 1.05–1.16, *p* = 0.0002). Similar to analyses with overall survival, the patients with lower albumin levels had an increased risk of progression or death compared to patients with higher albumin levels (HR: 1.76, 95% CI: 1.40–2.21, *p* < 0.001).

**Conclusion:** The available evidence demonstrates that albumin levels may be a prognostic biomarker in advanced cancer patients treated with ICIs. Further research is needed to delineate the role of albumin levels in patients treated with ICIs in the adjuvant setting, as well as the possible benefit of therapeutic approaches to improve hypoalbuminemia.

## Introduction

Immune checkpoint inhibitors (ICIs) have bocame a vital part of cancer care in the last decade (Darvin et al., 2018; Robert, 2020). First, ipilimumab, an antibody against CTLA-4, and later several monoclonal antibodies against PD-1 or PD-L1, have demonstrated improved survival in almost all tumors and across different treatment lines, either as monotherapy or in combination with chemotherapy or targeted therapy (Hodi et al., 2010; Rotte, 2019; Tan et al., 2021). These agents act by removing the immunosuppression created by the T-cell exhaustive checkpoints in the tumor microenvironment to aid the immune system in fighting tumors more efficiently (Waldman et al., 2020; Zhang et al., 2020). This mechanism of action, independent of specific targets on a tumor, permits ICI use in almost all tumors; however, at the same time, it creates a dependence on the tumor microenvironment for efficacy and, therefore, variable outcomes (Sadeghi Rad et al., 2021).

Despite the stunning rate of developments in therapeutic sites with ICIs, biomarker development has been relatively slow. Other than PD-L1 expression in non-small cell lung cancer (Pawelczyk et al., 2019), gastric cancer (Fuchs et al., 2018), and cervical cancer (Chung et al., 2019), and two tumor agnostic markers (microsatellite instability (Zhao et al., 2019) and tumor mutational burden (Huang et al., 2021)), no biomarker has consistently aided the decision-making process with ICIs. Several issues have factored into the slow biomarker development with ICIs, including the requirement of tissue samples and complex platforms (Guven et al., 2020; Lei et al., 2021; Rizzo et al., 2021). Furthermore, tissue-based biomarkers have not been able to reflect the status of the immune system, which is the main driver of ICI efficacy (Havel et al., 2019). Recently, peripheral blood-based biomarkers have garnered significant interest as the indirect indicators of host immune status, and a significant body of data has been generated with several compound biomarkers measuring the anti-tumor activity of lymphocytes and the uncontrolled inflammatory pressure of neutrophil, platelets, and monocytes, the neutrophil-lymphocyte ratio (NLR), the platelet-lymphocyte ratio (PLR) and the pan-immune-inflammation value (Petrova et al., 2020; Aktepe et al., 2021; Chen Y et al., 2021; Guven et al., 2021; Guven et al., 2022b).

Similar to peripheral blood-based biomarkers derived from blood count, albumin levels are significantly affected by inflammatory pressure (Soeters et al., 2019), and lower albumin levels are seen in cases of chronic inflammatory disorders and cancer as a negative acute phase reactant (Fiala et al., 2016; Schneider et al., 2022). Additionally, albumin levels are correlated with nutritional status, with lower albumin levels acting as an important denominator of malnutrition and poor general condition (Bharadwaj et al., 2016; Keller, 2019). Due to these features, albumin could be used as a prognostic biomarker in patients with cancer. Several studies in patients treated with chemotherapy, radiotherapy, or targeted therapy have demonstrated a higher risk of mortality or progression with lower albumin levels (Ikeda et al., 2017; Iede et al., 2022; Sun et al., 2022). Similarly, albumin levels could also be used for prognosis estimation in ICI-treated patients (Stares et al., 2021; Chen et al., 2022; Guo et al., 2022; Yoo et al., 2022). However, the data on the albumin in ICI-treated patients are limited, with heterogeneous patient cohorts, small sample sizes, and variable cut-offs. Therefore, we conducted a systematic review and meta-analysis to evaluate the association between survival outcomes and albumin levels in patients treated with ICIs.

## Methods

### Literature search

We conducted a systematic review following the Preferred Reporting Items for Systematic Reviews and Meta-Analysis guidance (PRISMA) (Page et al., 2021). The PRISMA checklist could be found in the supplement. This protocol was registered with the PROSPERO registry (Registration Number: CRD42022337746). We used the Web of Science, PubMed, and Embase databases to systematically filter the published studies from inception to 1 June 2022. The selected MeSH search terms were “albumin” OR “Glasgow prognosis score” OR “prognostic nutritional index” OR “albumin-globulin ratio” AND “immunotherapy” OR “immune checkpoint inhibitors” OR “immune-checkpoint inhibitor” OR “immune checkpoint blockade”. Both albumin and albumin-based compound prognostic markers were selected as search terms to prevent missing relevant studies.

### Study selection and data extraction

We included studies that met the following inclusion criteria: 1) prospective and retrospective studies to investigate the prognostic effects of albumin levels in ICI-treated patients with cancer; 2) articles reporting the hazard ratio (HR) for overall survival (OS) and/or progression-free survival (PFS); and 3) articles for which the full text was available in English. The exclusion criteria were as follows: 1) duplicated articles; 2) chapters of books, case reports, editorial letters, review articles, and opinion papers; 3) animal studies; 4) studies including patients without cancer; 5) studies with clinical endpoints other than PFS or OS; 6) studies without data for HRs and confidence intervals (CIs); and 7) studies without a predefined albumin cut-off to define hypoalbuminemia.

Two authors independently extracted the following data from the available studies (DCG, TKS) following the Meta-Analysis of Observational Studies in Epidemiology (MOOSE) guidelines (Stroup et al., 2000): lead author names, years of publication, study countries, tumor types, total numbers of patients, albumin cut-offs, and HRs with 95% CIs for OS and PFS. Due to the availability of studies in metastatic settings only, we collected PFS data for progression events. Any disagreements between the authors (DCG, TKS) during data collection were resolved by a discussion with the senior author (SY). The individual study qualities and risk of bias were evaluated independently by two authors (DCG, EE) using the Newcastle-Ottawa Scale (Wells et al., 2000).

### Meta-analyses

The study’s primary objective was to evaluate the association between OS or PFS and the presence of hypoalbuminemia in patients treated with ICIs. The secondary objective was to evaluate the association between the OS and the presence of hypoalbuminemia in subgroup analyses according to study location (United States, Europe, Far East), study sample size (<100, 100–200, >200), albumin cut-off (3.5 or other), and tumor type (NSCLC, urothelial cancer, GEC, MPM, HCC, basket cohorts).

We performed the meta-analyses with the generic inverse-variance method with a random-effects model due to the high degree of heterogeneity across the studies. The principal summary measure was HRs with 95% two-sided CIs, and the heterogeneity within each subgroup was reported with I-square statistics. Furthermore, we conducted additional analyses witha fixed-effects model after the exclusion of studies that caused a high degree of heterogeneity. The possibility of publication bias was assessed by the visual inspection of funnel plots. The meta-analyses were conducted with Review Manager software, version 5.4 (the Nordic Cochrane Center, the Cochrane Collaboration, Copenhagen, Denmark), and *p*-values below 0.05 were regarded as statistically significant.

## Results

### Eligible studies

Our systematic search retrieved a total of 2965 records. After removing the duplications (n = 1419), we screened the titles of the remaining 1546 records and excluded 1419 more records due to the following reasons: topic irrelevance (n = 944); review, case report, commentary, meta-analysis, or editorial (n = 379); animal studies (n = 70); and studies not in English (n = 26). We screened the titles and abstracts of the remaining 127 articles and excluded 91 records due to the lack of HRs for survival (n = 28) and the lack of separate reporting for albumin levels in studies evaluating albumin-based prognostic scores (n = 63). We evaluated the full texts of the remaining 36 articles and included these 36 studies in the meta-analyses. The PRISMA diagram for article selection is included in the supplement (Supplementary Figure S1).

### Study characteristics

Thirty-six studies encompassing 8406 patients were included in the meta-analyses. Almost half of the studies were conducted in NSCLC cohorts (n = 15), followed by urothelial (n = 5), gastroesophageal (n = 5), and hepatocellular cancers (n = 3) (Table 1). In addition, three studies included basket cohorts (Dercle et al., 2016; Bigot et al., 2017; Yoo et al., 2022). The sample sizes were variable and spanned from 44 (Ichiki et al., 2019) to 1714 (Yoo et al., 2022). More than half of the studies were reported from the Far East (n = 15) (Ichiki et al., 2019; Jiang et al., 2020; Kitadai et al., 2020; Takada et al., 2020; Ke et al., 2021; Morimoto et al., 2021; Ng et al., 2021; Pu et al., 2021; Sato et al., 2021; Tokuyama et al., 2021; Chen et al., 2022; Kim et al., 2022; Li et al., 2022; Wu et al., 2022; Zhang et al., 2022), followed by Europe (n = 12) (Dercle et al., 2016; Bigot et al., 2017; Mezquita et al., 2018; Svaton et al., 2018; Cantini et al., 2020; Awada et al., 2021; Onn et al., 2021; Ruiz-Bañobre et al., 2021; Stares et al., 2021; Assié et al., 2022; Schneider et al., 2022; Stares et al., 2022). A 3.5 gr/dL albumin cut-off was most frequently used in the included studies (n = 20) (Dercle et al., 2016; Bigot et al., 2017; Mezquita et al., 2018; Formica et al., 2020; Jiang et al., 2020; Kitadai et al., 2020; Lee et al., 2020; Takada et al., 2020; Awada et al., 2021; Brown et al., 2021; Khaki et al., 2021; Morimoto et al., 2021; Ng et al., 2021; Onn et al., 2021; Pu et al., 2021; Ruiz-Bañobre et al., 2021; Sato et al., 2021; Stares et al., 2021; Tokuyama et al., 2021; Assié et al., 2022; Chen et al., 2022; Kim et al., 2022; Li et al., 2022; Schneider et al., 2022; Stares et al., 2022; Wu et al., 2022; Yoo et al., 2022; Zhang et al., 2022), while eight studies evaluated the effect of albumin levels on survival by using albumin as a continuous measure (Svaton et al., 2018; Ichiki et al., 2019; Cantini et al., 2020; Swami et al., 2020; de Kouchkovsky et al., 2021; Ke et al., 2021; Abuhelwa et al., 2022; Guo et al., 2022). All but two studies reported HRs for OS (Stroup et al., 2000; Schneider et al., 2022; Zhang et al., 2022), while data for PFS was available in 15 studies (Stroup et al., 2000; Svaton et al., 2018; Jiang et al., 2020; Kitadai et al., 2020; Takada et al., 2020; Brown et al., 2021; de Kouchkovsky et al., 2021; Morimoto et al., 2021; Ruiz-Bañobre et al., 2021; Chen et al., 2022; Kim et al., 2022; Schneider et al., 2022; Stares et al., 2022; Wu et al., 2022; Yoo et al., 2022; Zhang et al., 2022). Most studies tested prognoses in patients treated with ICI monotherapy, and nivolumab was the most commonly used ICI in the studies (n = 21) (Table-1). Most studies had a low or intermediate risk of bias according to the Newcastle-Ottawa Scale (Table 2).

**TABLE 1:** Baseline characteristics of included studies.

Lead author, year	Country	Sample size (n)	Tumor type	IO type	Albumin cut-off value (g/dL)	Outcomes	Analysis model (univariate/Multivariate)	Adjustment factors	Additional comments
Dercle L, 2016, Dercle et al. (2016)	France	251	Basket	-Anti-PD-1 (n = 145)	3.5	OS	Multivariate	-SMI < 53 cm2 m2	-PS3-CT score calculated using high TB, low SMI(53 cm^2^ m2) and non-pulmonary visceral metastases allows to identify patients with prolonged OS on anti-PD-1/-L1 therapy, independent of conventional prognostic scores
				-Anti-PD-L1 (n = 106)				- TB RECIST >9 cm	
								-NPVM	
								-High LDH	
								- >2 metastasis	
								-ECOG > 0	
Bigot F, 2017, Bigot et al. (2017)	France	155	Basket	-Anti-PD1 (n = 64)	3.5	OS	Multivariate	-High LDH	-The Gustave Roussy Immune Score, based on albumin, LDH and NLR, allows a better selection of patients for ICT phase I trials
				-Anti-PD-L1 (n = 64)				-NLR > 6	
				-Anti-GITR (n = 23)				-Albumin	
				-Anti-PD-L1 + anti-CSF1R (n = 2)					
				-Anti-PD1 + anti-CD137 (n = 2)					
Svaton M, 2018, Svaton et al. (2018)	Czech Republic	120	NSCLC	Nivolumab	Continuous	-PFS	Univariate	-N/A	-A significantly shorter PFS and OS was evident in patients with lower hemoglobin concentration and higher calcium level corrected for albumin
						-OS			
Mezquita L, 2018, Mezquita et al. (2018)	France and Spain	466	NSCLC	-Anti-PD-1 (n = 382)	3.5	-PFS	Multivariate	-Age	-Pretreatment lung immune prognostic index, combining dNLR greater than 3 and LDH greater than ULN, was correlated with worse outcomes for ICI.
				-Anti-PD-L1 (n = 66)		-OS		-Smoking History	
				-Anti-PD-L1 + Anti- CTLA4 (n = 18)		-DCR		- Histologic subtype	
								-Lines of ICIs	
								-Number of metastatic sites	
								-ECOG	
								-LDH	
								-NLR	
								-Albumin	
Ichiki Y, 2019, Ichiki et al. (2019)	Japan	44	NSCLC	-Nivolumab (n = 26)	Continuous	-PFS	Multivariate	-Agent	-There was no significant difference in the prognosis between nivolumab and pembrolizumab
				-Pembrolizumab (n = 18)		-ORR		-Pathological type	
						-OS		-ECOG	
								-PET (SUV)	
								-WBC	
								-NLR	
								-LDH	
								-Albumin	
Lee CS, 2020, Lee et al. (2020)	United States	106	NSCLC	-Nivolumab (n = 59)	3.5	-PFS	Multivariate	-Weight loss upon starting ICI	-Significant weight loss (>5%) prior to starting ICI were significantly associated with OS (HR: 2.48, 95% CI: 1.31–4.68, *p* = 0.0052)
				-Pembrolizumab (n = 25)		-OS		-Age	
				-Atezolizumab (n = 21)		-IRAE		-Albumin	
				-Avelumab (n = 1)					
Swami U, 2020, Swami et al. (2020)	United States	169	Cutaneous Melanoma	Anti-PD-1 based therapies	Continuous	-PFS	Multivariate	-Brain Metastasis	-Contrary to some prior studies NLR, Platelet count, BMI, radiation, and antibiotics were not associated with PFS or OS.
						-OS		-Liver Metastasis	
								-Albumin	
Jiang M, 2020, Jiang et al. (2020)	China	76	NSCLC	-Nivolumab (n = 59)	4.3	-PFS	Multivariate	-Gender	-A higher PLR prior to the fifth dose of ICIs was also a prognostic biomarker, which significantly correlated with shorter OS in both the durvalumab (*p* = 0.028) and nivolumab cohorts (*p* = 0.046)
				-Durvalumab (n = 17)		-OS		-Age	
						-RR		-ECOG	
								-Smoking History	
								-Line of Treatment	
								-Albumin	
Takada K, 2020, Takada et al. (2020)	Japan	226	NSCLC	-Nivolumab (n = 131)	3.5	-Overall response	Multivariate	-ECOG	-PD-L1 tumor proportion score ≥50%, dNLR ≥2.79, lymphocyte-monocyte ratio <2.12, and red blood cell distribution width ≥15.9% were independent predictors of both PFS and OS.
				-Pembrolizumab (n = 95)		-DCR		-History of radiation therapy	
						-PFS		-NLR	
						-OS		-LMR	
								-RDW	
								-Albumin	
Cantini L, 2020, Cantini et al. (2020)	Netherlands	107	MPM	Nivolumab	Continuous	-PFS	Multivariate	-Platelet count	-High absolute monocyte count was significantly associated with worse PFS (HR: 3.16, 95% CI: 1.56–6.37, *p* = 0.001)
						-OS		-Neutrophils	
						-ORR		-Albumin	
Kitadai R, 2020, Kitadai et al. (2020)	Japan	215	NSCLC	-Nivolumab (n = 125)	3	-Overall response	Multivariate	-Liver metastasis	- Patients with liver metastasis who has good Royal Marsden Hospital (0–1) and Gustave Roussy Immune (0–1) scores showed significantly longer OS ((HR: 0.37; 95% CI: 0.16–0.84) and PFS (HR: 0.46; 95% CI: 0.22–0.97)
				-Pembrolizumab (n = 64)		-PFS		-ECOG	
				-Atezolizumab (n = 26)		-OS		-Driver mutation	
								-Albumin	
Formica V, 2020, Formica et al. (2020)	United Kingdom	57	mGOJ/GC	-Pembrolizumab (n = 26)	3	-OS	Multivariate	-CRP	-Gastric Inflammatory Prognostic Index, combining NLR, CRP, and albumin, is the first inflammatory index with a significant prognostic value in patients with mOGJ/GC receiving ICIs
				-Nivolumab (n = 16)				-NLR	
				-Avelumab (n = 15)				-Albumin	
NG YYK, 2020, Ng et al. (2021)	Singapore	114	HCC	-PD-1/PD-L1 inhibitor monotherapy (n = 67)	2.8	-PFS	Multivariate	-Bilirubin	- Inferior OS was found to be independently associated with higher bilirubin levels (HR: 6.82; 95% CI: 1.47–31.72), presence of diuretic-refractory ascites (HR: 44.46; 95% CI: 11.01–179.59), and HBV-associated HCC (HR: 2.01; 95% CI: 1.12–3.60)
				-CTLA-4 inhibitor monotherapy (n = 4)		-OS		-Ascites	
				- ICI-ICI combination (n = 10)		-ORR		-Hep-B status	
				-ICI-locoregional combination (n = 22)		-DCR		-Albumin	
				- Other combinations (n = 11)		-IRAE			
Awada G, 2021, Awada et al. (2021)	Belgium	183	Melanoma	Pembrolizumab	3.5	-Overall response	Multivariate	-Brain metastasis	-Total metabolic tumor volume is a more comprehensive baseline biomarker than CRP, LDH, or ALC in predicting the futility of pembrolizumab
						-PFS		-Number of affected organs	
						-OS		-CRP	
								-ALC	
								-NLR	
								-Albumin	
Brown JT, 2021, Brown et al. (2021)	United States	53	Urothelial Cancer	-Nivolumab (n = 3)	3.8	-PFS	Univariate	N/A	-Baseline Modified Glasgow Prognostic Score of 2 was significantly associated with worse PFS (HR 3.91; CI, 1.74–8.82; *p* < 0.001) and OS (HR 6.37; CI, 2.46–16.48; *p* <.s001)
				-Pembrolizumab (n = 11)		-OS			
				-Atezolizumab (n = 39)					
Ke L, 2021, Ke et al. (2021)	China	120	Advanced Lung Cancer	N/A	Continuous	-OS	Univariate	N/A	- SUVmax ≥11.42 and LDH ≥245 U/L were associated with shorter OS (*p* = 0.001 and *p* = 0.004, respectively)
						-Best treatment response			
						-CB			
Sato S, 2021, Sato et al. (2021)	Japan	278	Gastric Cancer	Nivolumab	3.5	-PFS	Multivariate	-CRP	-C-reactive protein level of ≤0.5 mg/dl (HR = 0.476, *p* < 0.001), irAE occurrence (HR = 0.544, *p* < 0.001), performance status 0 (HR = 0.711, *p* = 0.028), lymphocyte count >1000/μL (HR = 0.686, *p* = 0.027), and differentiated histological type (HR = 0.740, *p* = 0.046) were independently associated with improved survival
						-OS		-irAE occurrence	
								-ECOG	
								-Lymphocyte count	
								-Platelet count	
								-Neutrophil count	
								-Albumin	
Ruiz-Banobre J, 2021, Ruiz-Bañobre et al. (2021)	Spain	119	Urothelial Cancer	-Nivolumab (n = 7)	3.5	-OS	Multivariate	-ECOG-PS (≥2 *versus* 0–1)	-Use of proton-pump inhibitors was associated with poor OS (HR = 1.83, 95% CI, 1.11–3.02; *p* = 0.02) and PFS (HR = 1.94, 95% CI, 1.22–3.09; *p* = 0.005), and lower DCR (OR = 0.38, 95% CI, 0.17–0.89; *p* = 0.03) and ORR (OR = 0.18, 95% CI, 0.02–1.60; *p* = 0.002)
				-Pembrolizumab (n = 29)		-PFS		- Metastatic sites	
				-Atezolizumab (n = 80)		-DCR		- Lymph node metastases	
				- Durvalumab (n = 3)		-ORR		-Liver metastases	
								-Bone metastases	
								-Brain metastases	
								-Peritoneal metastases	
								-LDH	
								-Albumin	
								-Hemoglobin	
								-NLR	
								-PPI use	
								-Antibiotic	
Stares M, 2021, Stares et al. (2021)	United Kingdom	230	NSCLC	-Pembrolizumab (n = 167)	3.5	-OS	Univariate	N/A	-A positive dynamic change was associated with favorable OS compared to patients whose 12-week albumin remained <3.5 g/dl (*p* = 0.011)
				-Chemo-immunotherapy (n = 63)					
Khaki AR, 2021, Khaki et al. (2021)	United States	357	Advanced Urothelial Cancer	-Pembrolizumab (n = 189)	3.5	-OS	Multivariate	-ECOG-PS (≥2 *versus* 0–1)	- A new risk score was created based on The Eastern Cooperative Oncology Group performance status, albumin,NLR, and liver metastases, with a higher score indicating a lower overall survival rate
				-Atezolizumab (n = 137)				-Albumin	
				-Nivolumab (n = 16)				-Hemoglobin	
				-Durvalumab (n = 11)				-ANC	
				-Avelumab (n = 1)				-NLR	
				-Unknown (n = 3)				-Liver metastases	
								-Bone metastases	
								-Platelet count	
Tokuyama N, 2021, Tokuyama et al. (2021)	Japan	45	Advanced gastric Cancer	Nivolumab	3.5	-OS	Multivariate	-Gender	-Glasgow Prognostic Score of 0 was significantly associated with better overall survival than those with scores of 1 or 2 (16.4 vs 4.2 months; *p* = 0.0006)
						-PFS		-Age	
						-ORR		-ECOG	
								-Ascites	
								-Peritoneal metastasis	
								-GPS	
Pu D, 2021, Pu et al. (2021)	China	184	NSCLC	-Pembrolizumab (n = 98)	3.5	-OS	Multivariate	-Gender	-Pretreatment AEC, AMC, ALB, NLR, and PLR are independent predictors for survival in advanced NSCLC patients treated with PD-1 inhibitors
				-Nivolumab (n = 86)		-PFS		-Age	
						-DCR		-Smoking History	
						-ORR		-Previous radiotherapy	
								-NLR	
								-PLR	
								-LDH	
								-ANC	
								-ALC	
								-AEC	
								-AMC	
Onn A, 2021, Onn et al. (2021)	Israel	453 (Albumin level available 374)	NSCLC	-Nivolumab (n = 176)	3.5	-OS	Multivariate	-Gender	-Radiotherapy regimens such as a total dose of 30–40 Gy may synergize with ICIs whilst a total dose of less than 10 Gy, a fraction size of 4.1–8 Gy, and irradiation of bone lesions may result in antagonistic effect with ICIs
				-Pembrolizumab (n = 139)				-Age	
				-ICI-chemotherapy combination (n = 101)				-ECOG	
				-Atezolizumab (n = 32)				-Line of Treatment	
				- ICI-ICI combination (n = 5)				-NLR	
								-ICI type	
								-Radiotherapy site	
								-Total radiotherapy dose	
								-Fraction size	
Morimoto K, 2021, Morimoto et al. (2021)	Japan	203	NSCLC	-Pembrolizumab + chemotherapy	3.5	-OS	Multivariate	-PD-L1 expression (<%50 vs. ≥ 50%)	-In patients with NSCLC, Pembrolizumab combined with platinum and pemetrexed, but not nab-paclitaxel/paclitaxel, resulted in shorter PFS and OS in elderly patients, compared with the same regimen in non-elderly patients
						-PFS		- Chemotherapy regimen	
						-DCR			
						-ORR			
de Kouchkovsky I, 2021, de Kouchkovsky et al. (2021)	United States	119	Urothelial Cancer	-Pembrolizumab (n = 81)	Continuous	-OS	Multivariate	- Histologic subtype	-In a group of aUC patients treated with an ICI who had genetic data available, the presence of a TERT promoter mutation was an independent predictor of better OS.
				-Atezolizumab (n = 35)		-PFS		-visseral metastases	
				-Nivolumab (n = 2)		-ORR		-ECOG	
				-Durvalumab (n = 1)				-BMI	
								-Hemoglobin (10 g/dl > vs l ≥ 10 g/dl)	
								-NLR	
								-TERT promoter mutation	
								- CDKN2B mutation	
Zhang Z, 2022, Zhang et al. (2022)	China	101	HCC	-Tislelizumab (n = 23)	3.5	-PFS	Multivariate	-BCLC Stage (B/C/D)	- IINS as a composite score of CRP, lymphocyte and albumin could be a useful prognostic score for patients with HCC receiving anti-PD-1 therapy
				-Camrelizumab (n = 69)		-DCR		-Albumin	
				-Pembrolizumab (n = 7)		-ORR		-Extrahepatic metastasis	
				-Toripalimab (n = 2)				-CA199 (U/ml) (≤18.31:>18.31)	
								-Cycles of anti-PD−1	
								-Combined with target therapy (no/yes)	
								-IINS (low vs high)	
Scheider MA, 2022, Schneider et al. (2022)	Germany	139	NSCLC	-Pembrolizumab + chemotherapy (n = 57)	4.42	-PFS	Multivariate	-Age	-A panel of HP and CP could be utilized as a risk stratification tool of PFS in patients with NSCLC receiving PD-1/PD-L1 checkpoint inhibitor
				-Pembrolizumab (n = 35)				-Gender	
				-Nivolumab (n = 22)				-Histology	
				-Atezolizumab (n = 14)				-Stage	
				-Durvalumab (n = 10)				-Line of Treatment	
				-Durvalumab + chemotherapy (n = 1)				-Target treatment (PD-L1 vs PD-1)	
								-Albumin	
Assie JB, 2022, Assié et al. (2022)	France	109	MPM	Nivolumab	3.5	-OS	Multivariate	-MPM histology	-Second-line nivolumab is effective In patients with malignant pleural mesothelioma in real-word settings. Hypoalbuminemia and patients beyond the age of 70 were associated with reduced effectiveness of nivolumab
						-PFS		-LIPI	
						-DCR		-Albumin	
						-ORR		-Best response	
								-Age	
Chen L, 2022, Chen et al. (2022)	China	146	Gastric Cancer	N/A	3.5	-OS	Multivariate	-Prealbumin	-The CONUT score including serum albumin, total cholesterol level and total lymphocyte count may be used as a risk stratification tool for survival in patients with gastric cancer receiving ICIs
						-PFS		-CEA (<2.54 vs ≥2.54)	
								-CA199 (<14.40 vs ≥14.40)	
								-CA724 (<2.56 vs ≥2.56)	
								-CONUT (score≤0 vs >0)	
								-Albumin	
								-Radical resection (R0 vs non R0)	
								-Surgery	
								-TNM stage	
								-Lauren type (intestinal vs diffuse + mixed + unknown)	
								-PD-L1 (negative + unknown vs positive)	
								-PD-1 (negative + unknown vs positive)	
								-Treatment (ICIs vs chemotherapy)	
Kim JH, 2022, Kim et al. (2022)	Korea	60	ESCC	-Nivolumab (n = 48)	3.5	-OS	Univariate	N/A	-Recent use of antibiotics, low PNI (<35.93), high mGPS (≥1), and increase in NLR after one cycle from baseline were significantly unfavorable factors for both PFS and OS.
				-Pembrolizumab (n = 12)		-PFS			
						-DCR			
						-ORR			
Li Y, 2022, Li et al. (2022)	China	261	HCC	-Pembrolizumab (n = 40)	3.5	-OS	Univariate	N/A	-HCC-GRIm-Score- a new tool integrated AST-to-ALT ratio and TBIL to GRIm-Score based on three objective variables, namely, NLR, serum albumin level, and LDH may have higher predictive value in identifying HCC patients who would benefit from ICIs therapy
				-Nivolumab (n = 5)					
				-Toripalimab (n = 128)					
				-Sintilimab (n = 67)					
				-Tislelizumab (n = 4)					
				-Camrelizumab (n = 17)					
Yoo SK, 2022, Yoo et al. (2022)	United States	1714	Basket	-PD-1/PD-L1 (n = 1422) -CTLA-4 (n = 7) -Combo (n = 285)	3.7	-PFS	Multivariate	-Albumin	-Pretreatment serum albumin is a robust predictor of radiographic response and survival and when combined with TMB, it may help improve patient stratification
						-OS		-NLR	
						-DCR		-TMB	
						-ORR		-FCNA	
								-Age	
								-Gender	
								-BMI	
								-Stage	
								-Line of Treatment	
								-Performance status	
								-Cancer type	
								-Drug class (CTLA-4 vs. PD-1/PD-L1 vs. combo)	
Guo Y, 2022, Guo et al. (2022)	United States	210	NSCLC	-Nivolumab (n = 19)	Continuous	-OS	Multivariate	-Age	-Hypoalbuminemia and reduction in albumin level were both risk factors of decreased OS in patients with NSCLC receiving ICIs monotherapy but not chemoimmunotherapy
				-Pembrolizumab (n = 90)				-Performance status	
				-Pembrolizumab + Chemo (n = 101)				-Pretreatment Albumin	
								-Albumin change	
								-Pretreatment NLR	
								-On treatment NLR	
								-On treatment PLR	
								-On treatment LDH	
Abuhelwa AY, 2022, Abuhelwa et al. (2022)	Europe, North America, and the Asia-Pacific region	429 (IMvigor210)	Urothelial Cancer	Atezolizumab	Continuous	-PFS	Univariate	N/A	-Addition of CRP to Bellmunt score including performans status, hemoglobin level, and the presence of liver metastasis may beter aid risk stratification of survival in patients with urothelial carcinoma receiving ICIs
(data from IMvigor210 and IMvigor211 clinical trials)		467 (IMvigor211)				-OS			
Wu Y, 2022, Wu et al. (2022)	China	101	NSCLC	N/A	3.54	-PFS	Multivariate	-Gender	-In multivariate analyses, high LDH and ECOG PS2 were linked to decreased OS whilst high albumin and any grade irAEs were linked to increased OS in patients with aNSCLC receiving ICIs
						-OS		-Age	
						-DCR		-Smoking history	
						-ORR		-Performance status	
								-Histological subtype (squamous, non-squamous)	
								-Clinical stage (IVA-IVB)	
								- Brain metastases	
								-Bone metastases	
								-Liver metastases	
								-EGFR mutation status	
								-PD-L1 expression (<%50 vs. ≥ 50%)	
								-Line of Treatment	
								-Thoracic radiotherapy	
								- irAEs	
								-Treatment type	
								-LDH	
								-Albumin	
								-NLR	
								-PLR	
								-SII	
								-SIRI	
Stares M, 2022, Stares et al. (2022)	Scotland	219	NSCLC	Pembrolizumab	3.5	-PFS	Multivariate	-Gender	-In patients with NSCLC receiving first-line pembrolizumab, The Scottish Inflammatory Prognostic Score (SIPS) composite score of albumin and neutrophil count, may be used to predict survival
						-OS		-Age	
								-Performance status	
								-Histological subtype (squamous, non-squamous)	
								-WCC (≤11 × 109/L, >11 × 109/L)	
								-Neutrophils (≤7.5 × 109/L, >7.5 × 109/L)	
								-NLR (≤5, >5)	
								-PLR (≤180, >180)	
								-Prognostic nutritional index (<45, ≥45)	

HR, hazard ratio; CI, confidence interval; RECIST, response evaluation criteria in solid tumors; LDH, lactate dehydrogenase; ECOG, eastern cooperative oncology group; CT, computed tomography; CRP, creactive protein; SMI, skeletal muscle index; RMH, royal marsden hospital; NPVM, non-pulmonary visceral metastases; OS, overall survival; PS7, 7-pt prognostic; TB, tumor burden; PD1, programmed cell death protein 1; PD-L1, programmed cell death ligand one; GITR, glucocorticoid-induced tumor necrosis factor receptor; CSF1R, Colony stimulating factor 1 receptor; CD137, cluster of differentiation 137; NLR, neutrophil-to-lymphocyte ratio; DCR, disease control rate; PET, positron emission tomography; SUV, standardized uptake value; LMR, lymphocyte-monocyte ratio; PFS, progression-free survival; PLR, platelet-lymphocyte ratio; RDW, red blood cell distribution width; WCC, white cell count; NSCLC, non-small-cell lung cancer; aNSCLC, advanced non-small cell lung cancer; ICIs, immune checkpoint inhibitors; GOJ, gastro-esophageal junction; GC, gastric cancer; MPM, malignant pleural mesothelioma; EGFR, epidermal growth factor receptor, irAEs, immune-related adverse events; SII, systemic inflammation index; SIRI, systemic inflammation response index; mGPS, modified glasgow prognostic score; MTV, metabolic tumor volume; TLG, total lesion glycolysis; TMB, tumor mutational burden; FCNA, fraction of copy number altered genome; BMI, body mass index, CTLA-4, cytotoxic T-lymphocyte antigen 4; ESCC, Esophageal squamous cell carcinoma; AST-to-ALT, ratio, aspartate transaminase-to-alanine transaminase ratio; GRIm-Score, The Gustave Roussy Immune Score, TBIL; total bilirubin; CEA, carcinoembryonic antigen; CA, 19–9, cancer antigen 19–9,CA72-4; cancer-related antigen 72–4, CONUT, score; controlling nutritional status score, LIPI; lung immune prognostic index, CP; ceruloplasmin, HP; haptoglobin, IINS; inflammation-immunity-nutrition score, BCLC; Barcelona Clinic Liver Cancer, aUC; locally advanced or metastatic urothelial carcinoma (aUC), AEC, absolute eosinophil count; ALC, absolute lymphocyte count; AMC, absolute monocyte count; ANC, absolute neutrophil count.

**TABLE 2:** Newcastle-Ottawa scores of included studies (Note: A star system was used for allow a semi quantitative assessment of study quality. A study was awarded a maximum of four stars for the selection and three stars for exposure/outcome categories. A maximum of two stars were awarded for comparability).

Lead author, year	Selection	Comparability	Exposure/Outcome	Reference
Dercle L, 2016	****	**	***	Dercle et al. (2016)
Bigot F, 2017	****	**	***	Bigot et al. (2017)
Svaton M, 2018	****	**	**	Svaton et al. (2018)
Mezquita L, 2018	****	**	***	Mezquita et al. (2018)
Ichiki Y, 2019	***	*	**	Ichiki et al. (2019)
Lee CS, 2020	***	*	**	Lee et al. (2020)
Swami U, 2020	****	**	***	Swami et al. (2020)
Jiang M, 2020	***	**	***	Jiang et al. (2020)
Takada K, 2020	****	*	***	Takada et al. (2020)
Cantini L, 2020	****	*	**	Cantini et al. (2020)
Kitadai R, 2020	****	**	***	Kitadai et al. (2020)
Formica V, 2020	***	*	***	Formica et al. (2020)
NG YYK, 2020	****	*	***	Ng et al. (2021)
Awada G, 2021	****	**	***	Awada et al. (2021)
Brown JT, 2021	***	**	***	Brown et al. (2021)
Ke L, 2021	****	**	***	Ke et al. (2021)
Sato S, 2021	****	**	**	Sato et al. (2021)
Ruiz-Banobre J, 2021	****	**	***	Ruiz-Bañobre et al. (2021)
Stares M, 2021	****	*	***	Stares et al. (2021)
Khaki AR, 2021	****	**	***	Khaki et al. (2021)
Tokuyama N, 2021	***	*	***	Tokuyama et al. (2021)
Pu D, 2021	****	**	***	Pu et al. (2021)
Onn A, 2021	****	**	***	Onn et al. (2021)
Morimoto K, 2021	****	*	***	Morimoto et al. (2021)
de Kouchkovsky I, 2021	****	**	***	de Kouchkovsky et al. (2021)
Zhang Z, 2022	****	**	***	Zhang et al. (2022)
Scheider MA, 2022	****	**	***	Schneider et al. (2022)
Assie JB, 2022	****	*	***	Assié et al. (2022)
Chen L, 2022	****	**	***	Chen et al. (2022)
Kim JH, 2022	***	*	***	Kim et al. (2022)
Li Y, 2022	****	**	***	Li et al. (2022)
Yoo SK, 2022	****	**	***	Yoo et al. (2022)
Guo Y, 2022	****	**	***	Guo et al. (2022)
Abuhelwa AY, 2022	****	**	***	Abuhelwa et al. (2022)
Wu Y, 2022	****	**	**	Wu et al. (2022)
Stares M, 2022	****	**	***	Stares et al. (2022)

### The association between hypoalbuminemia and overall survival

**FIGURE 1:**
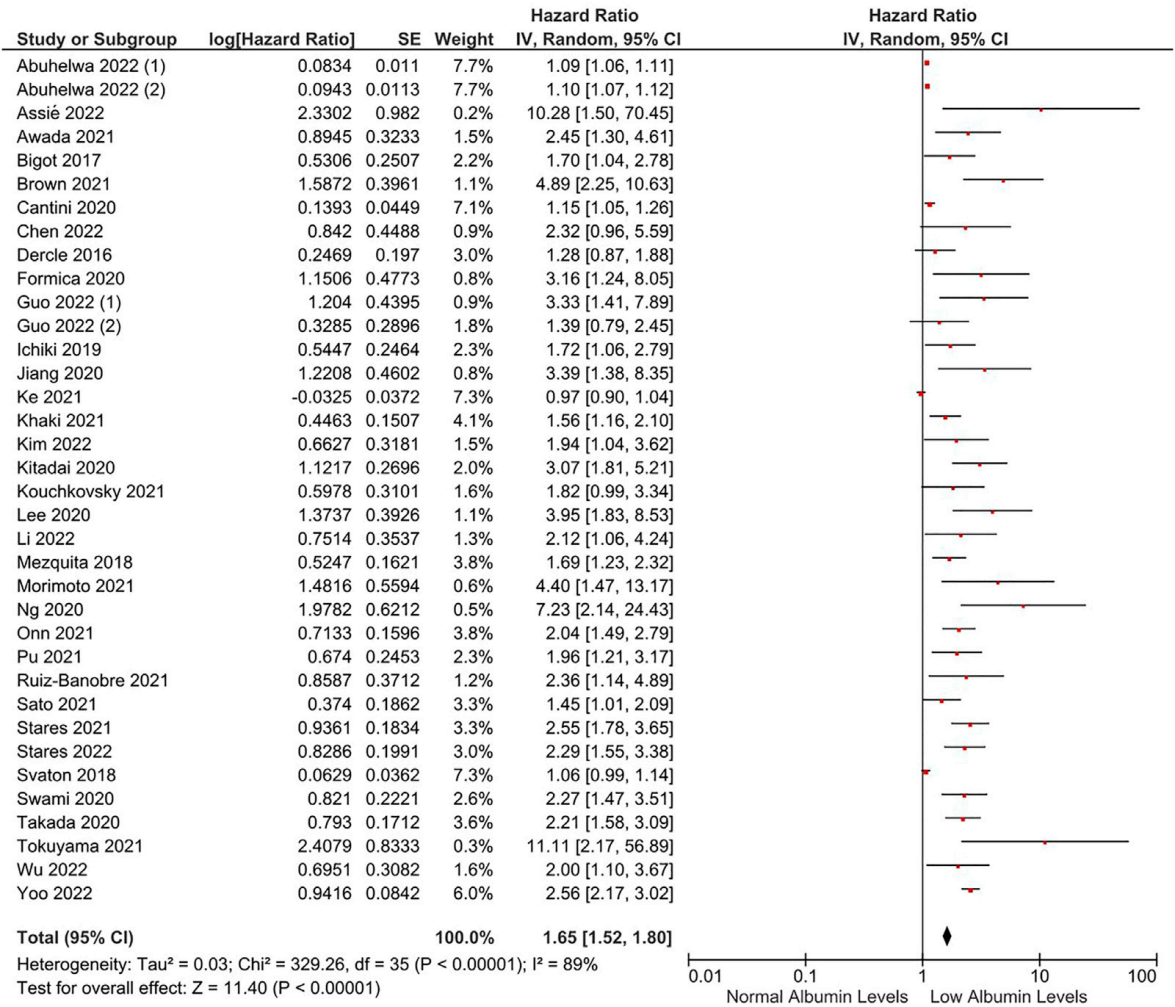
The association between albumin levels and OS. Lines (○) indicate 95% CIs. Diamond (♦) indicates the pooled effect size.

Thirty-four studies were included in the analyses for OS (Stroup et al., 2000; Dercle et al., 2016; Bigot et al., 2017; Mezquita et al., 2018; Svaton et al., 2018; Ichiki et al., 2019; Cantini et al., 2020; Formica et al., 2020; Jiang et al., 2020; Kitadai et al., 2020; Lee et al., 2020; Swami et al., 2020; Takada et al., 2020; Awada et al., 2021; Brown et al., 2021; de Kouchkovsky et al., 2021; Ke et al., 2021; Khaki et al., 2021; Morimoto et al., 2021; Ng et al., 2021; Onn et al., 2021; Pu et al., 2021; Ruiz-Bañobre et al., 2021; Sato et al., 2021; Stares et al., 2021; Tokuyama et al., 2021; Abuhelwa et al., 2022; Assié et al., 2022; Chen et al., 2022; Guo et al., 2022; Kim et al., 2022; Li et al., 2022; Stares et al., 2022; Wu et al., 2022; Yoo et al., 2022). In the meta-analysis, patients with lower albumin levels had a significantly increased risk of death (HR: 1.65, 95% CI: 1.52–1.80, *p* < 0.0001) than patients with higher albumin levels (Figure 1). The included studies had high degrees of heterogeneity (I^2^ = 89%). The important portion of the heterogeneity stemmed from the studies including albumin as a continuous parameter in the analyses. After the exclusion of these studies (n = 8) (Svaton et al., 2018; Ichiki et al., 2019; Cantini et al., 2020; Swami et al., 2020; de Kouchkovsky et al., 2021; Ke et al., 2021; Abuhelwa et al., 2022; Guo et al., 2022), heterogeneity was decreased to 47%, and a fixed-effect meta-analysis after the exclusion of these studies demonstrated a consistent negative effect of low albumin levels on OS (HR: 2.17, 95% CI: 1.99–2.36, *p* < 0.0001) (Supplementary Figure S2). Sensitivity analyses, with the exclusion of individual studies, also demonstrated a significantly higher risk of death in patients with lower albumin levels.

**FIGURE 2:**
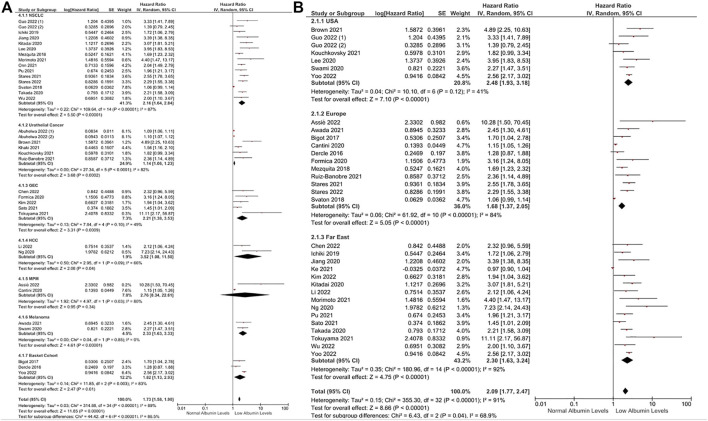
Subgroup analyses according to tumor types **(A)** and study location **(B)** in OS. Lines (○) indicate 95% CIs. Diamond (♦) indicates the pooled effect size.

**FIGURE 3:**
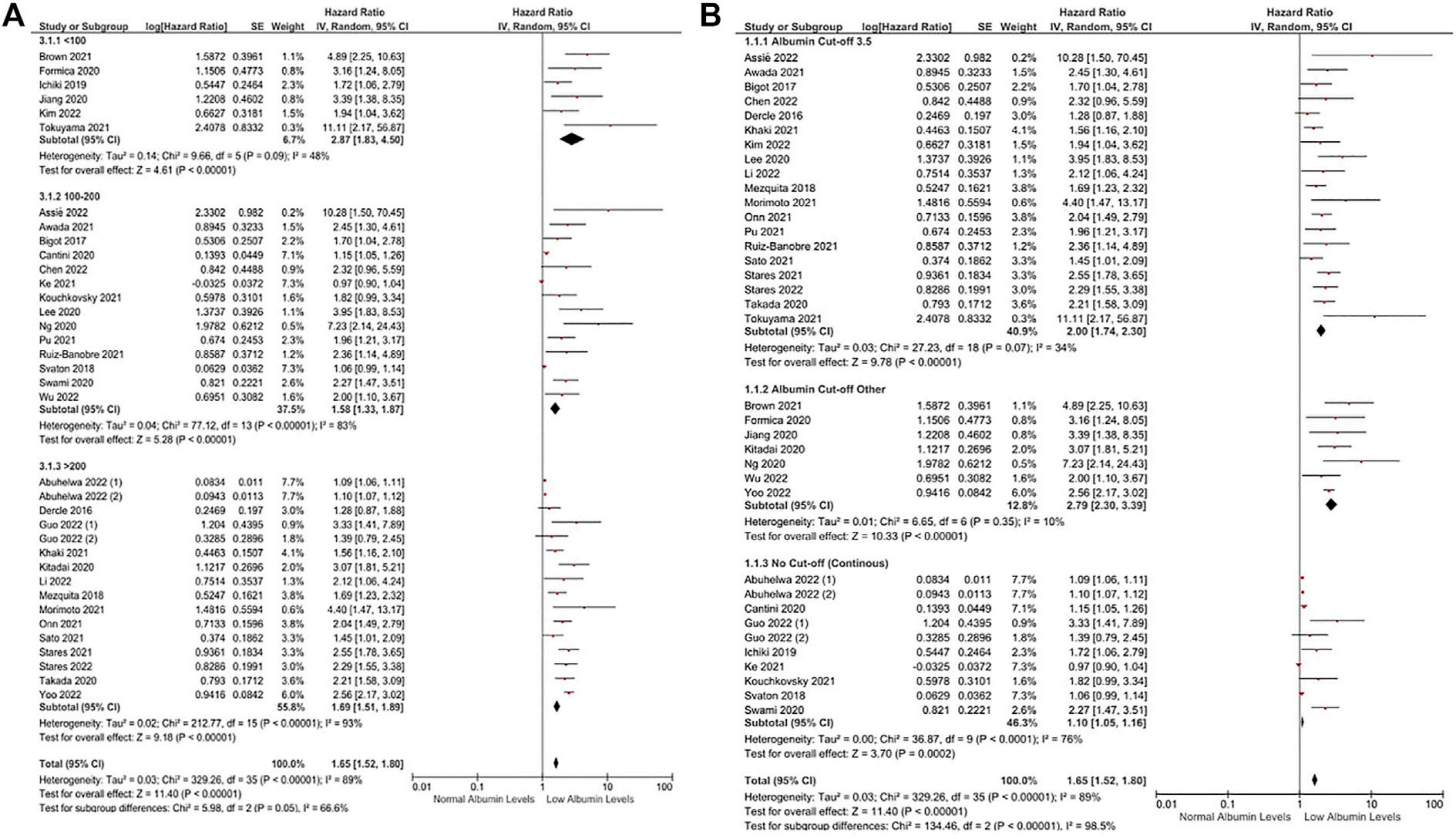
Subgroup analyses according to sample size **(A)** and albumin cut-offs **(B)** in OS. Lines (○) indicate 95% CIs. Diamond (♦) indicates the pooled effect size.

Subgroup analyses across several tumor types demonstrated a consistently higher risk of death in patients with lower albumin levels than in patients with higher albumin levels (Figure 2A). Similarly, subgroup analyses according to study location (HR: 2.48, 95% CI: 1.93–3.18, *p* < 0.0001 for the United States, HR: 1.68, 95% CI: 1.37–2.05, *p* < 0.0001 for Europe, and HR: 2.30, 95% CI: 1.63–3.24, *p* < 0.0001 for Far East) (Figure 2B) and sample size (HR: 2.87, 95% CI: 1.83–4.50, *p* < 0.0001 for sample sizes of <100, HR: 1.58, 95% CI: 1.33–1.87, *p* < 0.0001 for sample sizes between 100 and 200, and HR: 1.69, 95% CI: 1.51–1.89, *p* < 0.0001 for sample sizes over 200) (Figure 3A) demonstrated a negative association between lower albumin levels and OS. Additional subgroup analyses according to different cut-offs to define hypoalbuminemia demonstrated a significantly increased risk of death in patients with lower albumin levels (HR: 2.00, 95% CI: 1.74–2.30, *p* < 0.0001 for a cut-off of 3.5 gr/dL, and HR: 2.79, 95% CI: 2.30–3.39, *p* < 0.0001 for other cut-offs) (Figure 3B). Furthermore, in the subgroup analysis of eight studies using albumin levels as a continuous prognostic factor, every 1 gr/dL decrease in albumin levels was associated with a significantly increased risk of death by a factor of 10% (HR: 1.10, 95% CI: 1.05–1.16, *p* = 0.0002) (Figure 3B). The funnel plot evaluation demonstrated the possibility of publication bias, especially in studies with a sample size below 100 patients (Supplementary Figure S3).

### The association between hypoalbuminemia and progression-free survival

**FIGURE 4:**
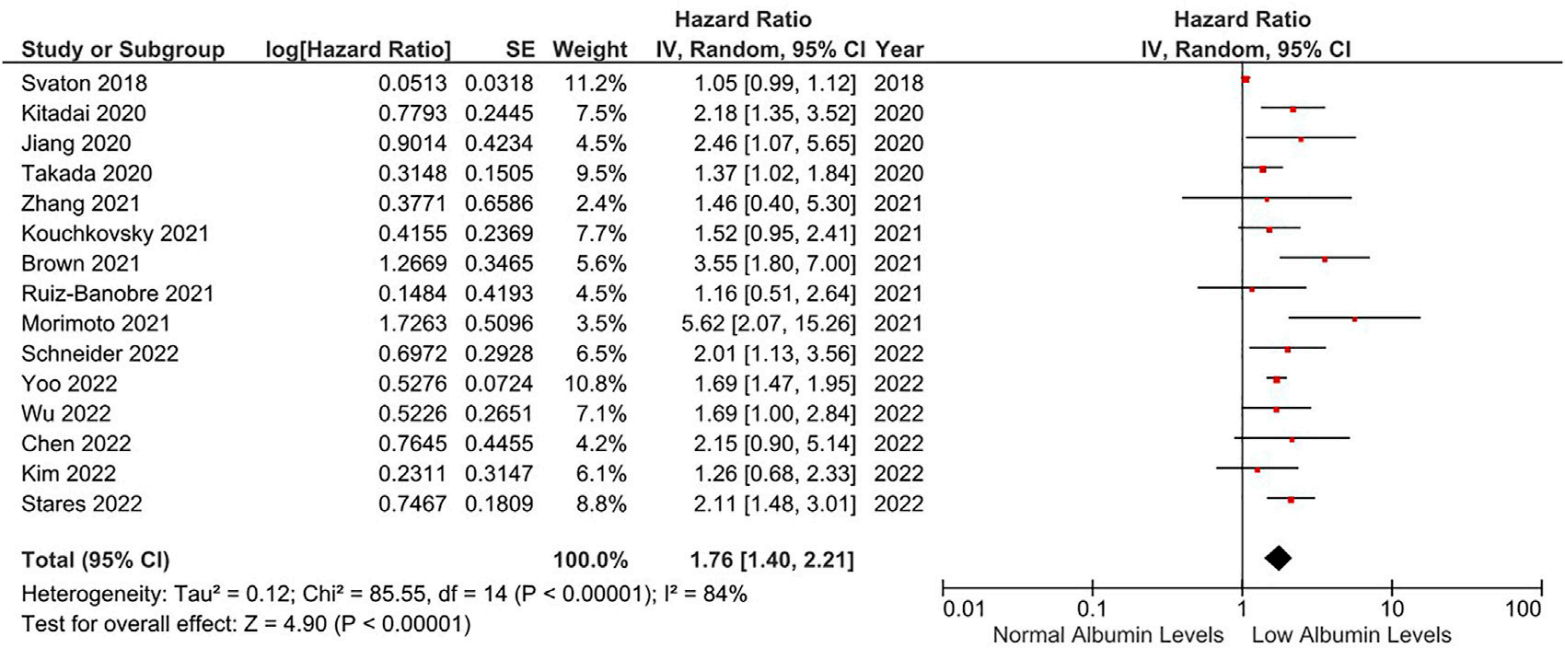
The association between albumin levels and PFS. Lines (○) indicate 95% CIs. Diamond (♦) indicates the pooled effect size.

Fifteen studies were included in the meta-analyses for PFS (Stroup et al., 2000; Svaton et al., 2018; Jiang et al., 2020; Kitadai et al., 2020; Takada et al., 2020; Brown et al., 2021; de Kouchkovsky et al., 2021; Morimoto et al., 2021; Ruiz-Bañobre et al., 2021; Chen et al., 2022; Kim et al., 2022; Schneider et al., 2022; Stares et al., 2022; Wu et al., 2022; Yoo et al., 2022; Zhang et al., 2022). Nine studies reported significantly lower PFS in patients with lower albumin levels, while the association between albumin levels and PFS did not reach statistical significance in the remaining six studies (Svaton et al., 2018; de Kouchkovsky et al., 2021; Ruiz-Bañobre et al., 2021; Chen et al., 2022; Kim et al., 2022; Zhang et al., 2022). In the meta-analysis of fifteen studies, patients with lower albumin levels had an increased risk of progression or death compared to patients with higher albumin levels (HR: 1.76, 95% CI: 1.40–2.21, *p* < 0.001) (Figure 4). The included studies had a significant degree of heterogeneity (I^2^ = 84%). Sensitivity analyses conducted by the subtraction of individual studies demonstrated consistent results. Heterogeneity was reduced to 31% after the exclusion of studies using albumin levels as a continuous measure in the analyses. An additional fixed-effect meta-analysis conducted after the exclusion of studies causing heterogeneity demonstrated a significantly increased risk of progression or death in patients with lower albumin levels (HR: 1.75, 95% CI: 1.58–1.95, *p* < 0.001) (Supplementary Figure S4). No additional subgroup analyses were conducted due to the limited number of studies evaluating the PFS.

## Discussion

In this meta-analysis, we observed a significantly increased risk or progression in ICI-treated patients with hypoalbuminemia. The adverse effect of low albumin levels in OS and PFS was consistent across tumor type, albumin cut-off for hypoalbuminemia, study country, and study sample size. To the best of our knowledge, the present study is the first meta-analysis evaluating the association between albumin levels and survival in patients treated with ICIs.

Nutritional status is an essential denominator of patients’ general conditions and is closely related to survival outcomes with anti-cancer treatments (Chen M.-F et al., 2021; Polański et al., 2021). Laboratory parameters like albumin are inexpensive and readily available measures of cancer patients’ nutritional status (Yan et al., 2021). In addition, albumin is a negative acute phase reactant, and its levels decrease in chronic inflammatory states like cancer (Khalil and Al-Humadi, 2020). While albumin production could be increased in the early stages of exposure to harmful insults like carcinogens, albumin production is significantly decreased in advanced cancer due to malnutrition and the inhibitory pressure of cytokines and chemokines, like C-reactive protein and interleukin-6 on the liver (Gupta and Lis, 2010; Svaton et al., 2018; Guo et al., 2022). Therefore, albumin levels could be a biologically plausible biomarker with the potential to reflect both the nutritional status and inflammatory pressure on patients with cancer and decreased levels of albumin could reflect a more advanced cancer stage and a more dismal prognosis. Furthermore, the measurement of albumin levels could be preferable to the readily available liver enzymes as a prognostic biomarker. The lower albumin levels were independently associated with the presence of cancer, while the levels of bilirubin, alanine transaminase, and aspartate transaminase were similar in patients with or without cancer in the pivotal Glasgow Inflammation Outcome Study (Proctor et al., 2010). Pavo et al. demonstrated that only albumin and butyrylcholinesterase levels were associated with all-cause mortality in cancer patients with non-liver primaries and patients without hepatic involvement (Pavo et al., 2017). These data support the robust role of albumin levels as a prognostic biomarker in patients with cancer compared to other candidate biomarkers related with hepatic function.

Several studies in patients treated with chemotherapy, surgery, targeted therapy, or radiotherapy demonstrated lower OS and PFS in cancer patients with lower albumin levels (Corcoran et al., 2015; Fan et al., 2017; Ikeda et al., 2017; Bekos et al., 2019). Furthermore, compound scores created by mixing albumin levels with positive acute reactants like CRP, as in the Glasgow prognostic score or globulin in albumin-globulin ratio, could also aid in prognosis estimations in patients with cancer and should be thoroughly investigated (Brown et al., 2021; Tokuyama et al., 2021; Guven et al., 2022a). In contrast, the data on the association between albumin levels and prognosis are still limited in ICI-treated patients. The available literature on ICI-treated patients is mainly focused on NSCLC cohorts and includes only patients with advanced-stage disease (Guo et al., 2022; Stares et al., 2022; Wu et al., 2022), while ICIs have become standard adjuvant treatment options for melanoma (Eggermont et al., 2018), NSCLC (O'Brien et al., 2022), bladder cancer (Bajorin et al., 2021), and esophageal cancer (Kelly et al., 2021). Additionally, most available studies on the prognostic role of albumin levels in ICI-treated patients have limited or absent data regarding previously established ICI-efficacy biomarkers like PD-L1 and tumor mutational burden levels (Davis and Patel, 2019; Xu et al., 2019; Huang et al., 2021). Therefore, additional data are needed to delineate the benefit of albumin levels for treatment selection in clinical practice and clinical trial stratification. Furthermore, available studies were significantly heterogeneous regarding albumin level cut-offs. They used various dichotomous cut-offs, as well as albumin levels as a continuous biomarker, limiting the generalizability of albumin levels as a biomarker (Jiang et al., 2020; Swami et al., 2020). Future studies should focus on the performance of variable, pre-defined cut-offs and the possible use of albumin levels as a continuous biomarker. Additionally, separate reporting for albumin levels as a continuous biomarker, in addition to dichotomous cut-offs, should be encouraged.

The present meta-analysis is subject to several limitations inherent to the methodology used and the characteristics of the included studies. First, we collected the reported HRs from the studies rather than conducting an individual patient data meta-analysis, so the presence of single-patient variables was not reported. The included studies were heterogeneous regarding tumor type, sample size, and albumin cut-offs, although we conducted subgroup analyses for these factors and observed consistent results. We did not conduct Bonferroni corrections for *p*-values, although the significance of most results was not expected to change, considering most of the analyses had a *p*-value of <0.0001. In addition, publication bias due to the higher publishing possibility of studies with positive results could not be excluded. Lastly, most of the included studies were conducted on patients treated with ICI monotherapy, while ICI-ICI, ICI-chemotherapy, and ICI-targeted therapy combinations have become standard care for most tumors, especially as first-line treatment (Mori et al., 2021; Xiong et al., 2021; Vafaei et al., 2022).

## Conclusion

In conclusion, the available evidence demonstrates that albumin levels could be a promising prognostic biomarker in patients with metastatic cancer treated with immunotherapy. Further research is needed to delineate the role of albumin levels in patients treated with ICIs in adjuvant settings and patients treated with ICI-based combinations, as well as the possible benefit of therapeutic approaches to improve hypoalbuminemia in ICI-treated patients.

## Data Availability

The original contributions presented in the study are included in the article/Supplementary Material, further inquiries can be directed to the corresponding author.
