# Supplementary material for: The association between albumin levels and survival in patients treated with immune checkpoint inhibitors: A systematic review and meta-analysis
Source: Front Mol Biosci. 2022 Dec 2;9:1039121. doi: 10.3389/fmolb.2022.1039121 (PMC9756377; doi:10.3389/fmolb.2022.1039121)
Supplement: Supplementary file 1 [file DataSheet1.docx]

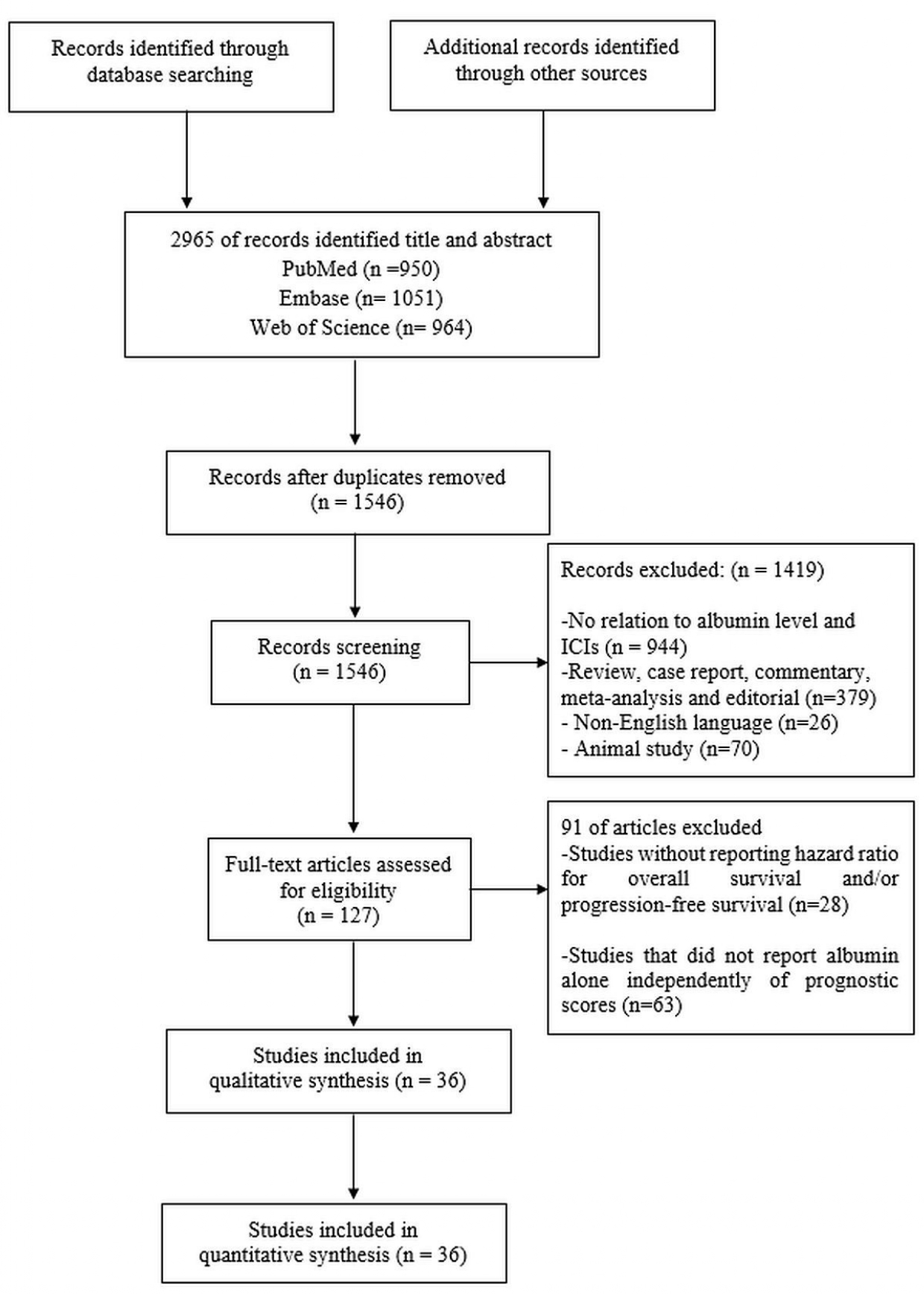


**Supplementary Figure S1.** PRISMA flow diagram


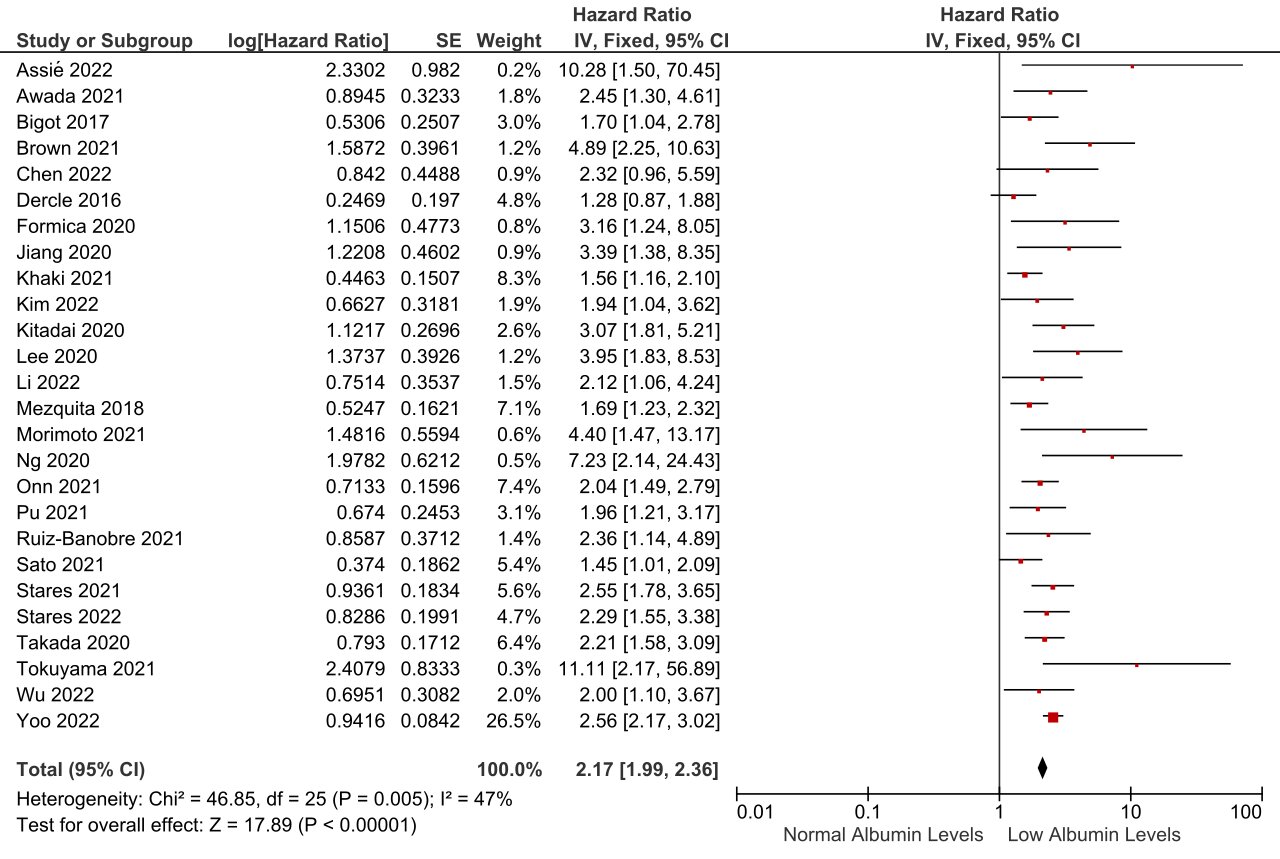


**Supplementary Figure S2.** Fixed meta-analysis for OS after the exclusion of studies using albumin levels as a continuous biomarker


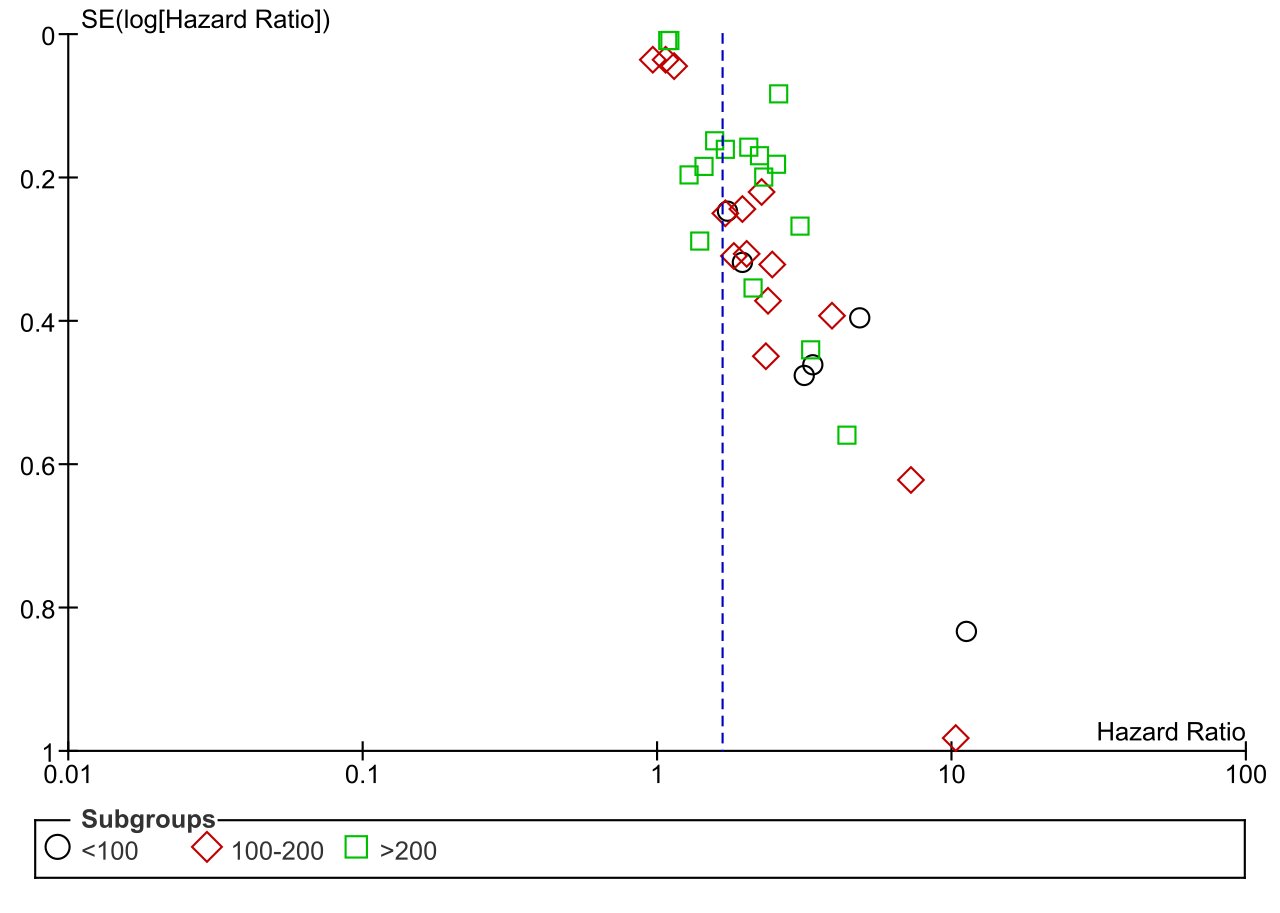


**Supplementary Figure S3.** Funnel plot for publication bias

**
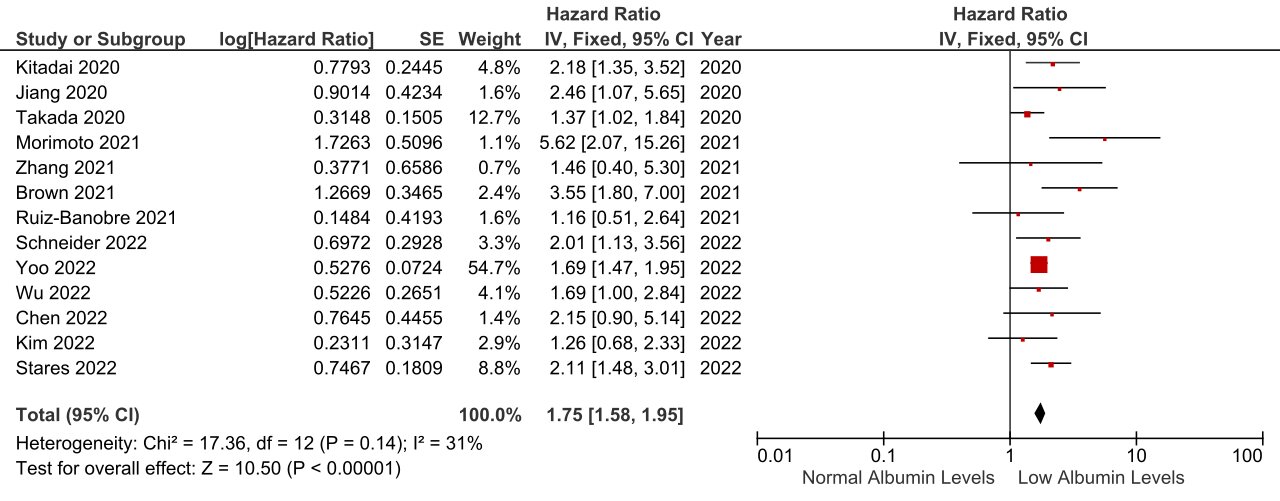
**

**Supplementary Figure S4.** Fixed meta-analysis for PFS after the exclusion of studies using albumin levels as a continuous biomarker
